# Surface hydrolysis-mediated PEGylation of poly(N-isopropyl acrylamide) based nanogels

**DOI:** 10.1093/rb/rbx022

**Published:** 2017-08-07

**Authors:** Jonathan T. Peters, Stanley Verghese, Deepak Subramanian, Nicholas A. Peppas

**Affiliations:** 1 McKetta Department of Chemical Engineering, The University of Texas at Austin, Austin, TX, USA; 2 Institute for Biomaterials, Drug Delivery, and Regenerative Medicine, The University of Texas at Austin, Austin, TX, USA; 3 Department of Biomedical Engineering, The University of Texas at Austin, Austin, TX, USA; 4 Department of Pediatrics, and Department of Surgery and Perioperative Care, Dell Medical School, The University of Texas at Austin, Austin, TX, USA; 5 Division of Pharmaceutics, College of Pharmacy, The University of Texas at Austin, Austin, TX, USA

**Keywords:** PNIPMAAm, hydrogels, PEG, passivation

## Abstract

In this work, poly(N-isopropyl acrylamide-co-acrylamide) [P(NIPAAm-co-AAm)] nanogels were modified by hydrolysis above the lower critical solution temperature (LCST) to localize carboxylic acid functional groups at the surface (surface hydrolysis). PNIPAAm copolymerized with 15% and 20% nominal AAm in the feed were prepared and compared to equivalent hydrogels with acrylic acid. The effect and extent of surface hydrolysis was confirmed by potentiometric titration and zeta potential. These surface modified nanogels were then modified with primary amine functionalized PEG chains. Surface hydrolysis-mediated PEGylation had little effect on the swelling response of the nanogels, while also preventing adsorption of model proteins in physiological relevant conditions. While both 15% and 20% AAm gels both decreased protein adsorption, only the 20% AAm gels resulted in fully preventing protein adsorption. The results presented here point to surface hydrolysis as a new route to passivate nanogels for use *in vivo.*

## Introduction

The use of nanoparticles as delivery vehicles for chemotherapeutics is beneficial as it allows for improved localization of the toxic therapeutics to the site of the cancerous tissue [1]. The application of nanoparticles to intravenous drug delivery depends greatly on the circulation half-life. This is necessary for particles of all types, as their increased size leads to removal from the blood stream via the reticular endothelial system (RES). 

Polymer surface modification with poly(ethylene glycol) (PEG) has been shown to be an effective means of increasing the circulation half-life [2]. However, systems based on functionalized amides, such as homo- and co-polymers of N-isopropyl acrylamide (NIPAAm), are stable preventing their reactivity with the functionalized PEGs available, requiring the addition of a reactive comonomer. However, the inclusion of these functional groups results in reduction of the temperature response. Often it introduces undesirable pH sensitivity [3]. Copolymerization with acrylamide (AAm) to form P(NIPAAm-co-AAm) nanogels prepared at a molar feed ratio with 15%–20% has shown that the presence of AAm increases the overall swelling response; at the same time the LCST was shifted from 32°C to 37–39°C [4].

The component AAm is susceptible to hydrolysis. Under extreme pH and temperature conditions AAm can be converted to AA in the presence of water. Under normal conditions this hydrolysis is not desirable, as it leads to AA distribution throughout the nanoparticle. The phenomenon of syneresis observed in crosslinked PNIPAAm can be used to protect the amide groups in the core of the particles. This protection is attained by the expulsion of water, thus preventing hydrolysis. By triggering the response and then exposing the hydrogels to an extreme pH level, the AAm unites near the surface of the nanogels can be localized (due to the increased concentration of water at the surface) [5].

Hoare and Pelton have proven this result with P(NIPAAm-co-AAm) containing 10% molar feed AAm Nominal (initial) concentration of the total monomer feed. In their studies, they demonstrated the relative hydrolysis of P(NIPAAm-co-AAm) gels performed at 30°C and 60°C and a range of pH conditions. Increasing temperature increased the rate and extent of hydrolysis. However, they proved that the increased temperature resulted in lower hydrolytic conversion. The effect of titration speed was further proof that there was a localization of the hydrolyzed groups near the surface of the nanogels. As the speed of the titration of the 30°C hydrolyzed system increased the concentration of carboxylic acid groups determined decreased. Comparatively, the 60°C hydrolysis sample showed no dependence on the rate of titration.

The dependence of the result based on the rate of titration shows that the diffusional timescale of the hydroxide ions into the nanogel limits the titration of the sample [5]. This demonstrated that the hydrolysis above the LCST concentrated hydrolysis to the surface of the nanogels. This conversion of AAm to surface localized acrylic acid provides a simple method/mechanism for PEGylation with an amine terminated PEG and EDC/NHS chemistry. This technique also lends itself to the concentration of PEG chains to the surface, limiting their impact on the overall swelling response of the nanoparticle.

## Materials and methods

### Nanogel synthesis

NIPAAm from Scientific Polymer, Ammonium Persulfate (APS), AA, AAm N’N’-Methylenebisacrylamide (MBAM), from Sigma Aldrich (St Louis, MO), and Sodium Dodecyl Sulfate (SDS) from Fisher Scientific (Pittsburgh, PA) were all used as received. In a standard reaction NIPAAm, the comonomer (AA or AAm), MBAM, and SDS were mixed together in 18.2 MΩcm water in the amounts specified in Table 1 below for the 20% comonomer nanogels. This solution was purged for 30 min at 70°C with nitrogen under 200 rpm stirring with a football shaped stir bar in a round bottom flask. Then APS dissolved in 18.2 MΩcm water was injected and the reaction was allowed to progress for 2 h under constant stirring. The reaction was then terminated by exposure to air. Nanogels not destined for surface hydrolysis were then purified against 18.2 MΩcm water for 2 weeks with twice daily water changes then freeze dried.

**Table 1:** Molar composition of nanogel synthesis for 20% AAm or AA systems

Component	mol % of total solution	% Monomer mol feed
NIPAAm	0.020%	71%
AAm or AA	0.0072%	20%
MBAM	9.0x10^−5^%	9%
SDS	1.9x10^−5^%	NA
Water	99.8%	NA

### Surface hydrolysis

Sodium hydroxide and hydrochloric acid from Fisher Scientific (Pittsburgh, PA) were used as received. Unpurified nanogel solution was heated to 60°C and then mixed in equal volumes with 1 N sodium hydroxide. This system was allowed to react for 3 days under constant stirring. The solution was sonicated at 60°C twice daily to break up aggregates. At the end of 3 days an equivalent volume of 1 N hydrochloric acid is added to bring down the temperature and pH simultaneously. The pH is then adjusted with 0.1 N hydrochloric acid to a pH of 7. The nanogel product was then dialyzed against 18.2 MΩcm water for 2 weeks with twice daily water changes then freeze dried.

### EDC/NHS PEG attachment

N-Hydroxysuccimide (NHS) and 1-Ethyl-3-(3-dimethylamiopropyl)carbodiimide (EDC) from Thermo Scientific (Waltham, MA), and NH_2_-PEG from Lysan Bio. (Arab, AL) were all used as received. In a standard reaction carboxylic acid containing particles were suspended in Borate buffer at a pH of 8.4 at a concentration of 4.7 mg/ml. EDC and NHS were suspended in Borate buffer at a concentration of 25 mM and amine-PEG was suspended at a concentration of 20 mM. Then 3 parts particle solution, 3 parts PEG solution, and 2 parts NHS solution are mixed. Two parts EDC solution is added and the solution is allowed to react overnight. It was then dialyzed against 18.2 MΩcm water for 1 week with twice daily water changes to remove unreacted PEG and excess buffer then freeze dried.

### Zeta potential and swelling measurements

Zeta potential and particle size were measured using a Malvern Zetasizer ZS (Malvern, UK). The effect of temperature on swelling size was collected by dissolving freeze dried particles in 1 ml DI water and adjusting the temperature by 3°C increments and then allowed to equilibrate for 60 s. Temperature was ramped 20°C–59°C–20°C to examine the effect of hysteresis on the samples particle size. Zeta potential was measured in 0.01 x PBS.

### pH titrations

Titrations were completed using an autotitrator (Hanna instruments HI902c, Carrolton, TX). A known mass of nanoparticles were suspended in 5 mM KCl. The samples were then auto-titrated with a 0.01 N NaOH solution. The exact normality of the titrant was determined by titration of a known mass of potassium hydrogen phthalate.

### TEM microscopy

Images were obtained on a Transmission Electron Microscope (FEI Tecnai, Hillsboro, OR) operating at 80 kV. Carbon coated copper grids (400 mesh) were plasma treated using an Emitech Glow Discharge instrument to render them hydrophilic prior to adding 5 µl of the nanoparticle suspension. After 30 s, the nanoparticle suspension was wicked off using Whatman 1 filter paper. The particles were negatively stained by placing a 5 µl drop of 2% uranyl acetate on the particle-coated grid for 30 s before being wicked off with Whatman 1 filter paper.

### Solution protein adsorption

To determine the effectiveness of the PEGylation reaction, these systems were incubated with model proteins, primarily Bovine Serum Albumin (BSA). Unmodified, AA, and PEGylated particles were incubated at 1 mg/ml in a 10 mg/ml BSA/1X PBS solution at 37°C and 45°C to compare the protein adsorption of swollen and collapsed particles. The particles and bound proteins were separated out via filtration through a 0.2 micron syringe filter, and their concentration left was determined by a micro BCA assay.

### QCM protein adsorption studies

Protein adsorption studies were performed on a dynamic Quartz Crystal Microbalance (dQCM) (Q4, Biolin Scientific, Stockholm, SW). In order to attach nanogels to the gold surface, first cysteine HCl was dissolved in a pH 8.4 borate buffer and passed over the surface to form a gold-sulfur bond. Then unattached cysteine was washed off with 1X PBS. Carboxylic acid containing monomers were then mixed with 25 mg of EDC and 25 mg of NHS in pH 8.4 borate buffer then immediately flowed over the cysteine coated gold surface. Particles modified with PEG were synthesized as in the EDC/NHS PEG Attachment section above, only including a 1:10 ratio of sulfur-PEG-NH_2_ (Laysan Biotech, Arab, AL) to the PEG-NH_2_, both MW 2000. These particles were bound by suspension in a Borate buffer, pH 8.4 then flowed over the gold surface. Unattached particles were washed off with 1X PBS. Particle modified surfaces then had a 10 mg/ml BSA solution in 1X PBS until saturation was obtained, signified by a constant Δf. Then 1X PBS was flowed to wash off BSA not adsorbed to the particles.

## Results and discussion

### Nanogel copolymerization synthesis and swelling response

**Figure 1:**
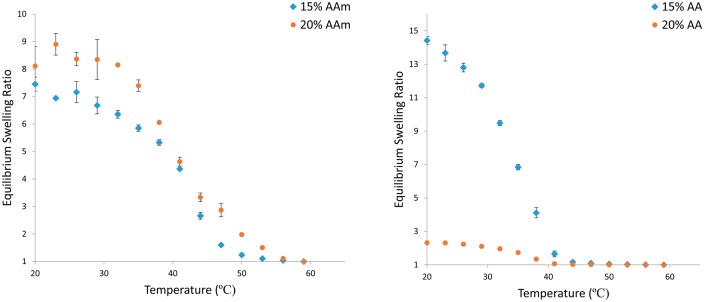
Equilibrium swelling ratio vs temperature of (**a**) P(NIPAAm-co-AAm) and (**b**) P(NIPAAm-co-AA) nanogels prepared at different total monomer molar feed percentages. 20–60 °C in DI water. Percentages are molar percentages of total monomer concentration in the feed (*N* = 3)

Nanogels composed of P(NIPAAm-co-AAm) and P(NIPAAm-co-AA) were successfully synthesized and purified. The paricles were spherical, and were a white powder when freeze dried. The swelling responses for these systems can be seen in Fig. 1 for AAm and AA, respectively, where equilibrium swelling ratio (ESR) is defined in equation 1.

$$ESR={\left(\frac{d}{d_{50{}^{\circ}C}}\right)}^{3}$$

Inclusion of AA at higher comonomer levels led to nanogels that demonstrated a greater reduction in the collapse experienced compared to copolymerization with AAm.

### Effect of surface hydrolysis

The relative concentrations of carboxylic acid groups in the nanogels were determined by potentiometric titration and are listed in Table 2. As seen in the work by Hoare and Pelton [5], we can calculate the rate at which titrant was added to reduce the change of voltage from 5 mV/min to 1 mV/min increased the result of AA containing nanogels by a factor of ∼1.5–2. Varying the rate of titration had a negligible effect on the result of the titration of the surface hydrolyzed nanogels. These results confirm the desired effect of the surface hydrolysis of the AAm based nanogels. The limited effect of titration speed on the surface hydrolyzed AAm gels show that the carboxylic acid groups are localized toward the surface as increased equilibration time is not necessary to overcome any mass transfer limitations into the nanogels.

**Table 2:** Diameter and concentration of carboxylic acid functional groups in AA and surface hydrolyzed AAm PNIPAAm comonomers

Polymer	Diameter	Expected Result	5mV/min	1mV/min Titration
	at 37˚C (nm)	(mol acid/g nanogel)	(mol acid/g nanogels)	(mol acid/g nanogels)
P(NIPAAm-co-AA)	250±10	1.3x10^−3^	3x10^−3^	6.5x10^−4^
15% AA				
P(NIPAAm-co-AAm)	434±3	NA	NA	NA
15% AAm				
P(NIPAAm-co-AAm)	410±7	4x10^−4^	4x10^−4^	5x10^−4^
15% AAm SH				
P(NIPAAm-co-AA)	199±6	1.8x10^−3^	1.3x10^−3^	1.9x10^−3^
20% AA				
P(NIPAAm-co-AAm)	561±1	NA	NA	NA
20% AAm				
P(NIPAAm-co-AAm)	292±1	4.7x10^−4^	1.1x10^−3^	1.02x10^−3^
20% AAm SH				

Expected values from feed ratios and assuming a 30% hydrolysis from surface hydrolysis.

The nanogels containing 15% AAm nominally in the reaction followed the trends observed by Hoare and Pelton [5], observing hydrolysis of 30% of the AAm in the nanogels [5]. The 20% AAm nanogels observed over twice the expected hydrolysis, however since the swelling response remains unaffected and the rate of titration no effect on the result it can be concluded that carboxylic acid groups are still localized toward the surface of the nanogels.

**Figure 2:**
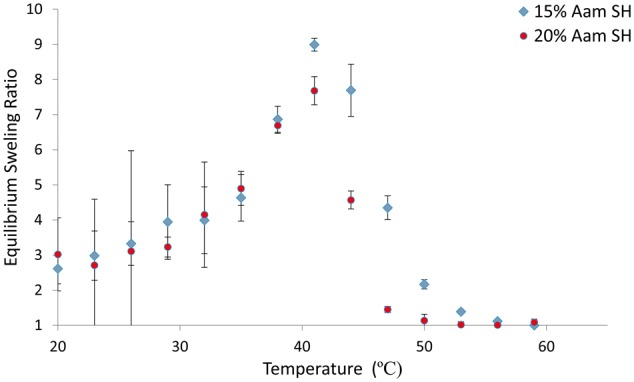
Equilibrium swelling ratio vs temperature of surface hydrolyzed P(NIPAAm-co-AAm) prepared with 15% and 20% AAm. 20–60 °C in DI water. Percentages are molar percentages of total monomer concentration in the feed (*N* = 3)

**Figure 3:**
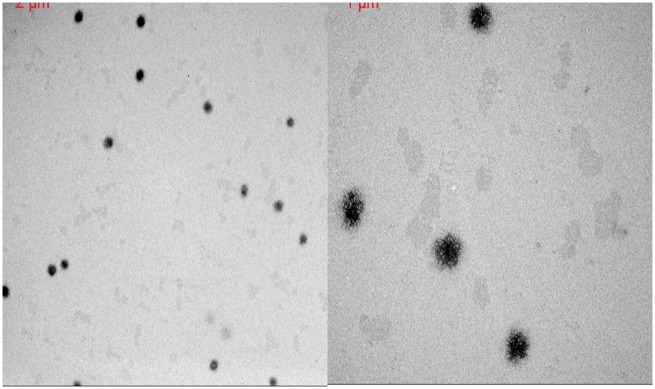
Characteristic TEM micrgraphs of unmodified (left) and surface hydrolyzed (right) 20% AAm, P(NIPAAm-co-AAm) nanogels. Percentages are molar percentages of total monomer concentration in the feed

The effect of surface hydrolysis on the swelling response can be viewed in Fig. 2. Both nanogel compositions exhibited a swelling response similar to their unmodified counterparts in Fig. 1 in the range of 37°C to 60°C, which represents the physiologically relevant temperature range these particles will experience *in vivo* (See Table 3). The surface hydrolyzed nanogels experience aggregation in temperatures below 37˚C causing large error in the DLS measurements. Representative TEM micrographs, seen in Fig. 3, show that surface hydrolysis had little impact on the structure of the nanogels. Images of both unmodified and modified nanogels show spherical particles of similar shape and size without the presence of containments.

**Table 3:** Size and concentration of carboxylic acid functional groups in surface hydrolyzed and PEGylated P(NIPAAm-co-AAm) comonomers

	Surface Hydrolyzed Diameter at 37˚C (nm)	Surface Hydrolyzed	PEGylated Diameter at	PEGylated
		(mol acid/g nanogel)	37˚C (nm)	(mol acid/g nanogel)
15% AAm	410 ± 7	4 x 10^−4^	384 ± 7	1.35 x 10^−4^
20% AAm	292.1 ± 0.85	1.1 x 10^−3^	533 ± 4	2 x 10^−4^

### PEGylation of surface hydrolyzed nanogels

**Figure 4:**
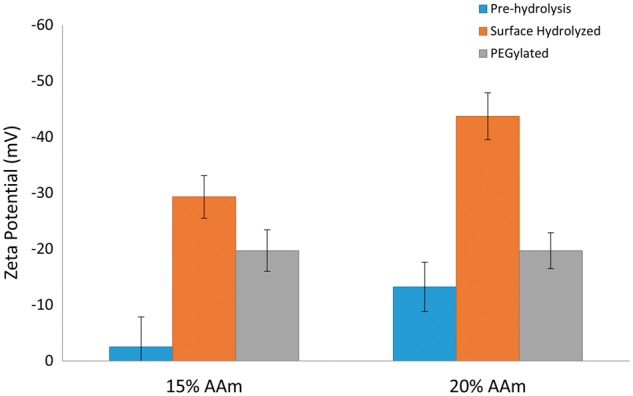
Zeta potential of P(NIPAAm-co-AAm) nanogels. Comparing surface charge of unmodified, surface hydrolyzed, and PEGylated nanogels in 0.1x PBS. Percentages are molar percentages of the total monomer feed (*N* = 3)

The results in Fig. 4 show the effect of surface hydrolysis on Zeta potential for 15% and 20% AAm formulations of surface hydrolysis and subsequent PEGylation. The low initial magnitude of Zeta potential demonstrates the neutrality of the P(NIPAAm-co-AAm) polymer. The magnitude of the Zeta potential greatly increases after the conversion of amide functional groups to anionically charged carboxylic acid groups. After PEGylation, the Zeta potential drops, showing that the PEG tethers are capping/shielding some of the charge of the present carboxylic acid groups present.

Comparing the results from potentiometric titration of the surface hydrolyzed and PEGylated nanogels quantifies the extent the PEGylation by the EDC/NHS route. PEGylation of the surface hydrolyzed 15% AAm nanogel caps 66% of the carboxylic acid groups. After PEGylation, the carboxylic acid functional groups get capped resulting in a decrease in overall swelling as a shell of non-temperature responsive PEG containing PNIPAAm groups gets functionalized. However, the LCST remains unchanged and the swelling response is still sufficient for drug delivery purposes.

### Cytotoxicity

**Figure 5:**
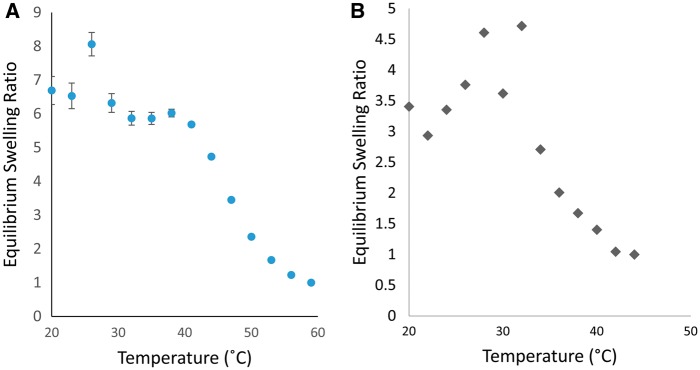
Equilibrium swelling ratio vs temperature of surface hydrolyzed 15% (**a**) and 20% (**b**) AAm P(NIPAAm-co-AAm) nanogels surface modified with 2000 MW PEG DLS. 20–60 °C in DI water (*N* = 3)

Macrophages are one of the primary cellular groups of the RES. Ensuring that these processes do not impart undesired toxic effects on these cell types is important to ensure that these particles do not resist uptake by killing immunologic cells. The relative toxicity, as measured by MTS assay are shown in Fig. 5 These results show that none of the PNIPAAm based nanogels show any appreciable toxic effects, as all particles are statistically similar to the positive control up to a concentration of 1 mg/ml.

### Protein adsorption studies

**Figure 6:**
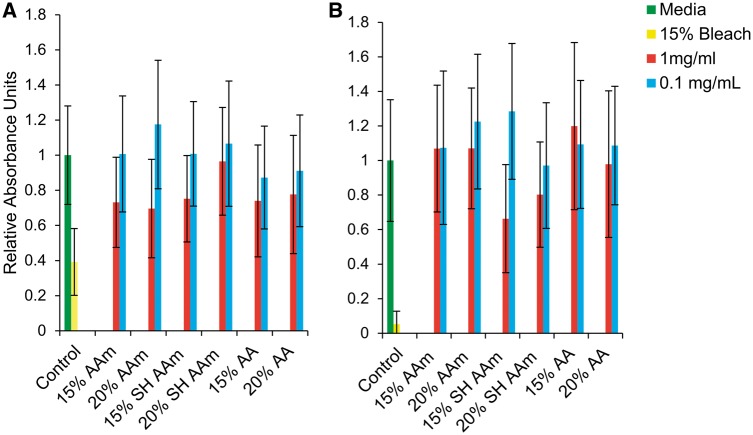
MTS Proliferation assay of P(NIPAAm-co-AAm) and P(NIPAAm-co-AA) nanogels with RAW 264.7 macrophages *N* = 4. (**a**) 2 h. (**b**) 24 h. Percentages are molar percentages of total monomer concentration in the feed (*N* = 4)

The adsorption of albumin to these particles is an important metric for determining the stealthing efficiency of PEGylation. Though there are many proteins that are responsible for coating foreign bodies in preparation of opsonization, albumin is one of the key proteins in this process. Fig. 6 shows the results of protein adsorption in solution. Though the PEGylated system does not show a statistically significant improvement over the unmodified nanogels, there is a slight improvement. This study also showed that the PEGylation of the 20% AAm nanogels did prevent a significant amount of BSA from adsorbing on the nanogels. Increasing the temperature to 45^°^C and collapsing the nanogels results in an increase in the adsorption of protein to all systems. This could be attributed to a number of factors, however it is likely caused by aggregation PNIPAAm nanogels experience in ionic liquids as demonstrated by Rasmusson et al. [5].

**Figure 7:**
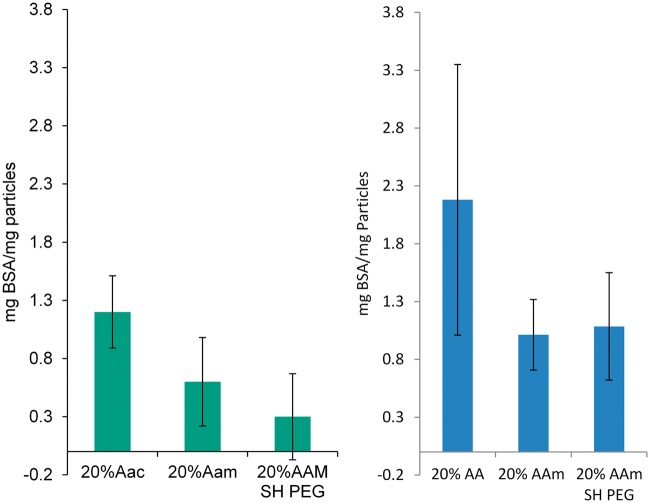
Bovine serum albumin (BSA) adsorbed to P(NIPAAm-co-AAm) and P(NIPAAm-co-AA) nanogels. We show results with two different sets of nanogels prepared in native form or after PEGylation

**Figure 8:**
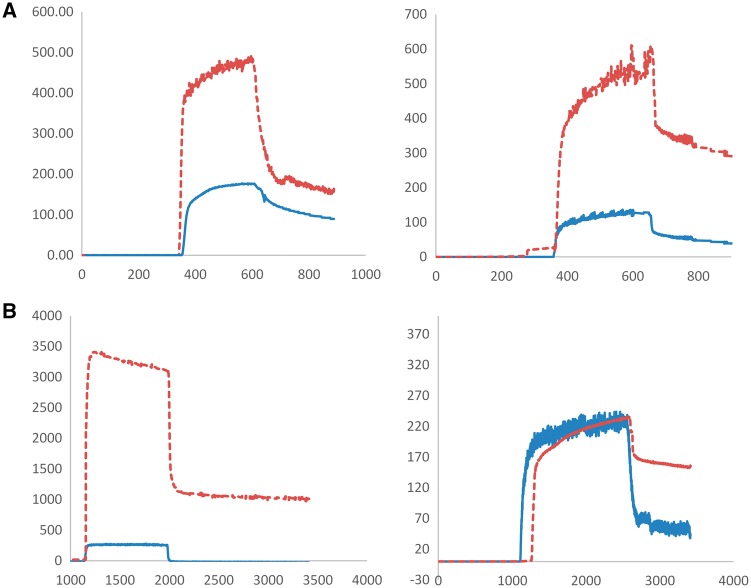
QCM Protein binding study of (**a**) P(NIPAam-co-AA) 15%AA and P(NIPAAm-co-AAm) 15% AAm surface hydrolyzed and PEGylated (**b**) P(NIPAAm-co-AA) 20% AA and P(NIPAAm-co-AAm) 20% AAm surface hydrolyzed and PEGylated. A solution of 10 mg/ml BSA was introduced at the 400 second mark then washed off. (dashed line– AA nanogels, solid line – PEGylated nanogels)

In order to elucidate the effect of peristaltic flow on the adsorption of BSA on the nanogels, dQCM was used to measure the strength of attachment in flow chambers. These studies; seen in Figs 7, 8 and Table 4, show that PEGylation of surface hydrolyzed acrylamide gels greatly reduces the adsorption of BSA to the nanogels as compared to their AA copolymer counterparts. The dQCM studies further confirm that the surface hydrolysis mediated PEGylation of the 20% AAm nanogels completely stealth the nanogels in physiological relevant conditions [6, 7].

**Table 4:** Bovine serum albumin (BSA) adsorbed to nanogels as determined by dQCM

Sample	Protein adsorbed 37˚C	Protein adsorbed 45˚C
	(kg/m^2^)	(kg/m^2^)
15% AA	162	292
15% AAm surface hydrolyzed and PEGylated	95	39
20% AA	1051	157
20% AAm surface hydrolyzed and PEGylated	0	53

Each sample observes higher levels of protein adsorption at 45˚C except the PEGylated 15% AAm nanogels. While the other three samples see a characteristic increase in protein adsorption attributed to the increase in temperature; the collapsed 15% AAm nanogels experienced a higher surface concentration of the PEG tethers better passivating the nanogels.

## Conclusions

The use of AA and AAm in copolymers has been tested as potential routes for incorporation of carboxylic acid functional groups. AAm units were proven to have less of an impact on the temperature response at relatively high comonomer concentrations than AA units, as seen in the temperature response swelling curves. The particles synthesized were spherical and stable in pure DI water.

The surface hydrolysis process was proven to have little to no effect on the structure of the nanogels, beyond what was intended. The particles remain structurally intact and the LCST and overall response to variations of temperature is unaffected. The 15% AAm follows the surface hydrolysis trend limiting the concentration of carboxylic acid and localizing them to the surface of the hydrogels. The 20% AAm nanogels deviate from this trend, seeing twice the hydrolysis expected. However, the carboxylic acid groups are still localized primarily near the surface of the nanogels.

PEGylation was confirmed with zeta potential measurements and by potentiometric titration. Protein binding studies were conducted in solution and under flow conditions. These studies showed surface hydrolysis mediated PEGylation effectively prevents protein adsorption to the nanogels. This shows that these systems should prove effective in an *in vivo* study to determine bioavailability and circulation half-life of this PEGylation scheme. This mechanism could also be adapted to other neutral-hydrolysable based systems to provide a noninvasive route for surface modification.

## Funding

National Institute of Biomedical Imaging and Bioengineering (NIBIB), the National Institutes of Health (NIH) (R21 EB012726-03A1).

*Conflict of interest statement*. None declared.
